# From epidemiology to molecular pathology: current research status and future prospects on the role of toxic metal exposure in the development and progression of bladder cancer

**DOI:** 10.3389/fcell.2026.1930640

**Published:** 2026-10-20

**Authors:** Danrui Li, Xu Sun, Tong Yu

**Affiliations:** 1 Operating Room 1, First Hospital of Jilin University, Changchun, China; 2 Operating Room 1, Second Hospital of Jilin University, Changchun, China

**Keywords:** biomarkers, bladder cancer, oxidative stress, precision prevention, signal transduction, toxic metals

## Abstract

Bladder cancer remains a prevalent and lethal urinary malignancy, characterized by high recurrence rates and persistently poor prognosis for muscle-invasive disease. This clinical reality underscores the urgent need to identify modifiable risk factors. Epidemiological evidence from case-control and prospective cohort studies has established dose-dependent associations with arsenic and consistent tissue-specific accumulation of cadmium and chromium, while lead remains conditional on metabolic status. Nevertheless, substantial study heterogeneity persists, largely due to differences in exposure assessment methods, dose-response patterns, and confounding by smoking. A comprehensive synthesis integrating population-level findings with mechanistic insights remains conspicuously lacking. This review addresses this gap by critically evaluating the epidemiological associations, appraising sources of heterogeneity, and synthesizing current mechanistic understanding. The evidence points to a convergent model in which these metals trigger ROS-mediated oxidative stress and mitochondrial dysfunction as upstream initiating events, which aberrantly activate two midstream signaling branches—the ROS-dependent p38-MAPK/NF-κB and PI3K/AKT pathways promoting proliferation and survival, and the ROS-independent SUMOylation/TGF-β/Smad2/3 axis driving epithelial-mesenchymal transition. NRF2 exhibits stage-dependent duality with extensive pathway crosstalk, and these signals converge on downstream outcomes, including resistance to apoptosis, invasion, and metastasis. Beyond cancer cell-intrinsic effects, emerging evidence indicates that metal-induced signaling aberrations also remodel the tumor microenvironment through immune reprogramming, stromal activation, and metabolic shifts. Based on these insights, we propose a biomarker-guided, multi-tiered prevention framework spanning primary environmental control, secondary chemoprevention, and tertiary targeted therapy. Despite encouraging progress, critical knowledge gaps persist, particularly regarding multi-metal co-exposure, population-level mechanistic validation, and clinical translation. This review identifies these limitations and charts interdisciplinary research directions that integrate advanced exposure assessment, multi-omics, and rigorous clinical evaluation to bridge environmental exposure science and precision prevention of bladder cancer.

## 1 Introduction

With an estimated 573,000 new cases and 213,000 deaths annually, bladder cancer remains a leading cause of cancer mortality worldwide, with 5-year survival rates for muscle-invasive disease stuck at 30%–50% despite therapeutic advances (Zhang Y. et al., 2023; Valencia Nieto et al., 2026). Its age-standardized incidence rates vary substantially across regions, reflecting differences in environmental exposures, lifestyle factors, and genetic susceptibility. Given that a significant proportion of bladder cancer cases are attributable to modifiable risk factors, identifying and effectively mitigating these exposures represents a critical strategy for reducing both incidence and mortality.

Tobacco smoking, occupational aromatic amine exposure, and arsenic contamination in drinking water are established risk factors for bladder cancer (Jahrreiss et al., 2020). The role of other environmental toxicants, particularly heavy metals, remains incompletely defined. Over the past 2 decades, a growing body of epidemiological evidence has implicated environmental exposure to toxic metals—particularly cadmium, chromium, lead, and arsenic—in the etiology of bladder cancer (Parida and Patel, 2023). Epidemiological and analytical studies have consistently reported significantly higher concentrations of these metals in the serum, urine, or tumor tissues of patients with bladder cancer than in healthy controls or adjacent non-cancerous tissues (Panaiyadiyan et al., 2023; Golabek et al., 2017; Chung et al., 2017; El-Agrody et al., 2024). For instance, cadmium accumulates preferentially in cancerous tissues. It correlates with lymph node positivity and advanced disease (Zhang L. et al., 2023), while chromium concentrations in bladder tumor tissues are markedly elevated relative to adjacent non-cancerous tissues. Arsenic shows the strongest dose-response relationship, with ecological and case-control studies consistently linking elevated drinking-water arsenic concentrations to increased bladder cancer risk (Pal et al., 2020; Chen et al., 2025). However, the epidemiological landscape remains controversial. Some high-quality prospective studies, such as the European EPIC Florence cohort, have reported no significant association between pre-diagnostic serum levels of multiple metals and bladder cancer risk among never and long former smokers without occupational exposure (Caini et al., 2025). These apparent discrepancies likely reflect fundamental differences in study design, exposure assessment matrices, and the temporal windows captured by different biological specimens—challenges that warrant critical evaluation.

From a mechanistic standpoint, the International Agency for Research on Cancer (IARC) has classified several toxic metals as definite or probable human carcinogens (Parida and Patel, 2023). Experimental studies have begun to delineate the molecular circuitry through which these metals drive bladder carcinogenesis. A convergent theme emerging from the literature is that cadmium, chromium, lead, and arsenic, despite their distinct physicochemical properties, have been associated with excessive production of reactive oxygen species (ROS) and mitochondrial dysfunction in experimental models (Zhang L. et al., 2023; Rej et al., 2026; Ali-El-Dein et al., 2025). These oxidative alterations are thought to contribute to the activation of oncogenic signaling cascades, including the p38-MAPK/NF-κB and PI3K/AKT pathways, which have been linked to increased cell proliferation, survival, and inflammatory signaling (El-Agrody et al., 2024; Ali-El-Dein et al., 2025). The discovery of a ROS-independent, SUMOylation-mediated TGF-β/Smad2/3 axis that drives epithelial-mesenchymal transition (EMT) adds another layer of mechanistic complexity (Yi et al., 2025). Adding further nuance, nuclear factor E2-related factor 2 (NRF2)—a master regulator of antioxidant defense—exhibits stage-dependent duality, conferring cytoprotection in early carcinogenesis but acquiring pro-tumorigenic properties in advanced disease (Xu et al., 2017). These interconnected perturbations ultimately converge on downstream cellular outcomes, including apoptosis resistance, enhanced invasion, and metastatic capacity (El-Agrody et al., 2024; Yi et al., 2025; Sawicka et al., 2020).

A critical gap remains in the current literature. No existing review has successfully bridged population-level epidemiological evidence with mechanistic insights into metal-induced molecular abnormalities. The epidemiological evidence itself is fraught with heterogeneity and confounding challenges. Critically, even where mechanistic understanding has advanced, it has not been translated into actionable prevention strategies. Most existing reviews address either epidemiological associations or molecular pathways in isolation, and few critically examine the translational implications and knowledge bottlenecks that impede clinical application. This lack of integration hampers biomarker-guided risk stratification, evidence-based environmental policy, and precision prevention for vulnerable populations.

To address this gap, the present review systematically synthesizes current evidence on the role of toxic metal exposure in bladder cancer development and progression, spanning the full spectrum from epidemiology to molecular pathology and translational applications. Specifically, we critically evaluate the epidemiological associations between exposure to cadmium, chromium, lead, and arsenic and bladder cancer risk, with particular attention to study heterogeneity, dose-response relationships, and the confounding role of smoking. We then analyzed this hierarchical mechanism framework. From upstream ROS-induced oxidative stress and mitochondrial dysfunction, through the activation of intermediate signaling networks, to downstream cellular effects, this process ultimately culminates in malignant transformation. Finally, we evaluate the translational potential of these mechanistic insights, outline a biomarker-guided, multi-tiered prevention strategy, and pinpoint the key knowledge gaps and interdisciplinary approaches needed to translate environmental exposure science into precision prevention of bladder cancer.

### 1.1 Search strategy and selection criteria

To ensure a systematic and reproducible literature synthesis, we conducted a comprehensive search of PubMed, Web of Science, and Scopus from inception to March 2026. The search strategy combined terms related to exposure (e.g., “toxic metals,” “heavy metals,” “cadmium,” “chromium,” “lead,” “arsenic”), the outcome (e.g., “bladder cancer,” “urothelial carcinoma”), and mechanisms. The primary mechanistic search terms focused on the most extensively studied pathways in metal carcinogenesis, including “oxidative stress,” “reactive oxygen species,” “signal transduction,” “apoptosis,” and “epithelial-mesenchymal transition.” To ensure broader coverage of the molecular pathology landscape, we additionally performed targeted searches using terms such as “tumor microenvironment,” “immune evasion,” “metabolic reprogramming,” “epigenetics,” and “DNA methylation.” Recognizing smoking as a major confounder and independent risk factor, we also incorporated epidemiological search terms including “smoking,” “smokers,” and “cigarette smoking” to capture studies that examined the interaction between metal exposure, smoking status, and bladder cancer risk. Finally, we manually screened reference lists of included articles and relevant reviews to identify studies examining less frequently investigated mechanisms. This hybrid approach—combining structured database searches with iterative reference screening—balanced focused mechanistic depth with comprehensive scope. We included original research articles (case-control, cohort, cross-sectional, and experimental studies in animal or cell models) published in English. We excluded reviews, editorials, letters, and conference abstracts. Two authors independently screened titles and abstracts, followed by full-text assessment. We resolved discrepancies by discussion with a third author. We also manually screened the reference lists of articles included and relevant reviews to identify additional studies. This rigorous approach allowed us to capture the breadth of evidence while maintaining methodological transparency.

## 2 Epidemiological evidence linking exposure to different types of toxic metals to the risk of bladder cancer

### 2.1 Association evidence across different exposure scenarios and major toxic metals

Toxic metal exposure is broadly classified as occupational or environmental. These differ substantially in concentration, duration, and co-exposure profiles. Occupational exposure occurs in mining, smelting, electroplating, and battery manufacturing. It typically involves high concentrations, prolonged durations, and well-defined routes. However, causal factors vary even within high-risk occupations. For instance, hard coal miners have elevated bladder cancer risk, but their urinary cadmium remains below occupational limits. This suggests that cadmium is not the primary driver in this cohort (Isermann et al., 2017). Environmental exposure occurs through contaminated water, air, or soil. It is characterized by low-dose, chronic, multi-metal co-exposure. In high-arsenic drinking water regions of West Bengal, India, arsenic levels in bladder tumors are markedly elevated. They are positively correlated with tumor aggressiveness and early recurrence (Pal et al., 2020). Similarly, soil contamination by arsenic, cadmium, and lead in a decommissioned petrochemical zone in southern China poses oral carcinogenic risks to children that exceed US EPA limits (Wang et al., 2020). These distinct scenarios offer complementary evidence for bladder cancer risk assessment. The following subsections first present the evidence for each metal individually, followed by a comparative synthesis that evaluates the relative strength and consistency of the evidence across metals and biological matrices.

Cadmium has been the most extensively investigated metal in relation to bladder cancer, with a convergence of evidence across multiple biological matrices. Case-control studies have consistently reported elevated cadmium concentrations in tumor tissues compared with adjacent non-cancerous specimens, a finding further corroborated by positive correlations between cadmium levels and lymph node involvement (Abdel-Gawad et al., 2016). At the systemic level, blood cadmium concentrations are significantly higher in MIBC patients and positively correlate with miRNA-21 expression, suggesting a potential role in aggressive phenotypes (El-Agrody et al., 2024; Awadalla et al., 2020). A large population-based NHANES analysis has extended these observations by showing a significant association between urinary cadmium and overall cancer risk (Ke et al., 2025). Although the research findings are consistent, the following points should be noted. First, the observed cadmium-cancer association may be confounded by smoking, as tobacco is a major source of cadmium exposure (Yi et al., 2025). Second, the cross-sectional design of most case-control studies precludes determining whether elevated cadmium levels precede or result from malignant transformation—a limitation that prospective cohort studies with pre-diagnostic biospecimens could potentially address (Zhang L. et al., 2023). Third, the dose-response relationship remains poorly quantified, and formal meta-analyses have not established a clear threshold or linear gradient specific to bladder cancer (Fanfani et al., 2024).

Collectively, evidence on cadmium remains consistent across tissue, blood, and urine matrices, supporting a positive association with bladder cancer. Median chromium concentrations in bladder tumor tissues are markedly higher than in adjacent non-cancerous tissues (Golabek et al., 2017). Hexavalent chromium promotes proliferation and migration while inhibiting apoptosis via regulation of Filamin A (Sheng et al., 2021). However, epidemiological findings are less uniform. Despite clear tissue enrichment, the EPIC Florence cohort found no significant association between serum chromium and bladder cancer risk in non-smokers without occupational exposure (Caini et al., 2025). Thus, chromium’s association is robust in tissue-based studies but inconsistent in systemic biomarker assessments. Lead shows a conditional association, with cross-sectional studies showing positive signals that BMI and nutritional status modify (Huang et al., 2025). Cross-sectional data alone cannot determine whether BMI directly modifies lead toxicity or whether unmeasured factors associated with both BMI and lead exposure drive the observed interaction. Prospective studies with repeated measurements are needed to clarify this effect modification. Elevated lead levels in cancer tissues correlate with altered expressions of Bax, IL-6, and AKT (Sawicka et al., 2020; Paithankar et al., 2021). Case-control studies consistently report higher blood and urine lead levels in patients than in controls (Panaiyadiyan et al., 2023). The evidence is broadly supportive but may be modified by metabolic and nutritional status.

Arsenic presents the strongest epidemiological evidence. Elevated drinking-water arsenic concentrations consistently increase bladder cancer risk. Arsenic ranks first in relative risk among all arsenic-induced cancers (Chen et al., 2025). In high-exposure areas, recurrent tumor tissues contain extremely high arsenic levels, closely linked to aggressive progression (Pal et al., 2020). Arsenic demonstrates the most robust dose-response relationship, supported by both ecological and case-control studies. A prospective nested case–control study within the Alpha-Tocopherol, Beta-Carotene Cancer Prevention (ATBC) cohort, which utilized pre-diagnostic toenail arsenic as an integrated biomarker of long-term low-level exposure, found no significant association with bladder cancer risk in Finnish male smokers (OR = 1.13, 95% CI: 0.70–1.81 for the highest vs. lowest quartile), suggesting that low-level arsenic exposure may not confer a substantial excess risk (Michaud et al., 2004).

Across the four metals, a distinct hierarchy of evidence emerges: arsenic shows the most robust dose-response relationship, followed by cadmium with consistent multi-matrix associations; chromium shows tissue enrichment but inconsistent serum-based findings; and lead remains the most conditional and susceptible to confounding by metabolic and nutritional factors. Notably, signal strength from tissue-based measurements is consistently higher than from serum or urine, highlighting the critical role of matrix selection in interpreting study heterogeneity (Table 1).

**TABLE 1:** Summary of epidemiological evidence linking exposure to major toxic metals with bladder cancer risk.

Metal	Exposure sources	Key epidemiological findings	Dose–Response relationship	Consistency
Cadmium (Cd)	Tobacco smoke, mining, smelting, battery manufacturing, contaminated food	Elevated levels in cancerous vs. non-cancerous tissues ( Abdel-Gawad et al., 2016 ); positive correlation with miRNA-21 in MIBC ( El-Agrody et al., 2024 ); NHANES: significant association with overall cancer risk (Ke et al., 2025); pooled RR = 1.38 (95% CI: 1.04–1.82) in the latest meta-analysis ( Deng et al., 2026 )	Limited formal dose–response meta-analysis (Fanfani et al., 2024); positive trend but not well-quantified	High across tissue, blood, and urine matrices
Chromium (Cr)	Electroplating, leather tanning, welding, contaminated water	Higher concentrations in tumor vs. adjacent tissues (Golabek et al., 2017); promotes proliferation/migration via Filamin A (Sheng et al., 2021)	No clear dose–response established	Robust in tissue-based studies; inconsistent in serum-based assessments (Caini et al., 2025)
Lead (Pb)	Mining, smelting, battery recycling, paint, contaminated soil	Independent association with bladder cancer risk (Huang et al., 2025); correlates with Bax, IL-6, and AKT expression (Paithankar et al., 2021)	Weak evidence; effect modification by nutritional status	Broadly supportive but conditional
Arsenic (As)	Contaminated drinking water (geogenic), mining, pesticides	High-dose exposure: most robust dose–response relationship (Deng et al., 2026); ranks first in relative risk among arsenic-induced cancers (Deng et al., 2026); correlates with tumor aggressiveness and recurrence (Pal et al., 2020); pooled RR = 1.60 (95% CI: 1.13–2.27) (Deng et al., 2026). Low-dose exposure: a prospective nested case–control study within the ATBC cohort, utilizing pre-diagnostic toenail arsenic as an integrated biomarker of long-term exposure, found no significant association in Finnish male smokers (OR = 1.13, 95% CI: 0.70–1.81 for highest vs. lowest quartile) (Michaud et al., 2004)	Nonlinear threshold ∼10 μg/L; increases steadily from 10 to 429 μg/L (Deng et al., 2026); null association observed at levels comparable to <100 μg/L, suggesting a potential threshold effect (Michaud et al., 2004)	Very high for high-dose ecological and case–control studies; emerging biomarker-based evidence suggests low-dose associations remain inconclusive (Michaud et al., 2004)

### 2.2 Study heterogeneity, dose-response relationships, and challenges in exposure assessment

Despite an overall positive trend, significant heterogeneity persists across studies. Some high-quality investigations, such as the European EPIC Florence cohort, report null associations between pre-diagnostic serum levels of multiple metals and bladder cancer risk in never and long former smokers without occupational exposure (Caini et al., 2025). In contrast, the US NHANES-based analysis found a significant positive association between urinary cadmium and overall cancer risk (Ke et al., 2025). These discrepancies are not mere statistical artifacts; they underscore fundamental differences in study design, population characteristics, and—most critically—the exposure time windows captured by distinct biological matrices.

A central but often underappreciated issue is the inherent temporal limitation of different exposure biomarkers. Serum and urinary metal levels primarily reflect recent or ongoing exposure (half-lives of days to weeks), whereas tissue concentrations represent cumulative, long-term bioaccumulation over years to decades (Zhang L. et al., 2023; Ali-El-Dein et al., 2025; Saini et al., 2025). This distinction is pivotal for interpreting epidemiological findings. The EPIC cohort used a single pre-diagnostic serum sample, which may completely miss the relevant exposure window for bladder carcinogenesis—a process with a latency period estimated at 20–40 years or more for arsenic (Zhao et al., 2022). By contrast, tissue-based studies consistently show metal enrichment in cancerous tissues and positive correlations with molecular markers of oxidative stress and pathway activation (Panaiyadiyan et al., 2023; Golabek et al., 2017; El-Agrody et al., 2024; Ali-El-Dein et al., 2025). This “matrix effect” likely explains why systemic biomarkers yield weaker or null associations in general populations, while tissue-based case-control studies yield stronger, more consistent signals. Future studies should prioritize repeated biological measures over time and incorporate specimens such as toenails and fingernails, which integrate exposure over several months (Salcedo-Bellido et al., 2021; Smith et al., 2022).

Beyond the matrix effect and exposure assessment challenges, another major challenge identified through our literature search is the complex role of smoking as a confounder and potential mediator. Another major challenge is the complex role of smoking. Tobacco smoke is a rich source of cadmium and lead, and smoking is an independent risk factor for bladder cancer (Jahrreiss et al., 2020; Chung et al., 2017; Isermann et al., 2017). This creates a challenging analytical dilemma: if cadmium and lead are mediators of smoking-related carcinogenesis, then conventional adjustment for smoking as a confounder would remove part of the true metal effect, leading to underestimation. Conversely, inadequate adjustment may overestimate the metal effect. Evidence for the mediation hypothesis is growing: a recent NHANES-based mediation analysis demonstrated that blood cadmium accumulation significantly mediates the association between smoking and all-cause and cancer-related mortality (Kim et al., 2023). For bladder cancer specifically, Long Interspersed Nuclear Element-1 (LINE-1) DNA methylation has been identified as a key mediator in smoking-related urothelial carcinoma risk (Jahrreiss et al., 2020). These findings imply that simple covariate adjustment for smoking status is insufficient to disentangle the independent effect of metals. Future analyses should adopt more sophisticated approaches, including causal mediation analysis, stratified analyses, and formal interaction tests to assess effect modification.

Dose-response relationships constitute a third layer of complexity. Although bladder cancer patients generally exhibit elevated metal levels, researchers have not uniformly established clear linear dose-response curves (Ali-El-Dein et al., 2025; Awadalla et al., 2020; Saini et al., 2025). A recent comprehensive systematic review and meta-analysis of 68 epidemiological studies provides the most up-to-date quantitative synthesis (Deng et al., 2026). Comparing the top and bottom tertiles of exposure, the pooled relative risks for bladder cancer were 1.60 (95% CI, 1.13–2.27) for arsenic and 1.38 (95% CI, 1.04–1.82) for cadmium (Deng et al., 2026). A nonlinear dose-response association was observed for drinking-water arsenic: it was not significant at or below 10 μg/L but increased steadily from 10 to 429 μg/L (Deng et al., 2026). For cadmium, evidence from a systematic review of 9 meta-analyses supports a causal role, but formal dose-response meta-analyses specific to bladder cancer remain limited (Fanfani et al., 2024). For lead, the evidence is less robust and may be modified by metabolic and nutritional status (Huang et al., 2025; Vagnoni et al., 2024). In real-world environments, metals coexist in complex mixtures, and synergistic or antagonistic interactions may further distort simple dose-response trends (Isermann et al., 2017). This underscores the critical need for future research to incorporate mixture-effect statistical models alongside refined exposure assessment strategies to elucidate true biological thresholds in the context of multi-metal interactions. Table 2 compares the advantages, limitations, and key findings associated with each biological matrix.

**TABLE 2:** Comparison of biological matrices for toxic metal exposure assessment in epidemiological studies.

Matrix	Exposure window	Advantages	Limitations	Key findings in bladder cancer
Serum/Blood	Recent (days–weeks)	Easy collection; standardized protocols	Reflects short-term exposure; single time-point may miss relevant window	EPIC cohort: null associations (Caini et al., 2025); Awadalla et al.: positive correlation with miRNA-21 (Awadalla et al., 2020)
Urine	Recent (days)	Non-invasive; reflects excretion	Subject to hydration status; short half-life	NHANES: positive association with cancer risk (Ke et al., 2025); reflects current body burden
Tissue (Tumor)	Cumulative (years–decades)	Direct target organ measurement; reflects long-term bioaccumulation	Invasive; limited sample availability; no pre-diagnostic collection	Consistently elevated Cd, Cr, Pb, and As in cancerous tissues (Panaiyadiyan et al., 2023; Golabek et al., 2017; Abdel-Gawad et al., 2016); correlates with oxidative stress markers (El-Agrody et al., 2024; Ali-El-Dein et al., 2025)
Toenails/Fingernails	Integrated (months)	Non-invasive; integrates exposure over several months; stable; suitable for prospective cohort studies with pre-diagnostic collection	Potential external contamination; slow growth rate	Successfully used in the ATBC cohort to assess long-term low-level arsenic exposure and bladder cancer risk (Michaud et al., 2004)

### 2.3 Causal inference: applying the Bradford Hill criteria to metal-bladder cancer associations

To move beyond a purely associative description of epidemiological findings and provide a robust foundation for the preventive strategies proposed later in this review, we adopt a structured causal inference framework. The Bradford Hill criteria, despite their subsequent refinements and critiques (Hill, 1965), remain the most widely recognized and practically applicable framework for assessing causation in environmental epidemiology. By systematically evaluating the evidence against these criteria—strength, consistency, specificity, temporality, biological gradient, plausibility, coherence, experiment, and analogy—we transform the scattered observational data into a graded causal determination. This graded framework, in turn, provides the essential rationale for prioritizing intervention strategies: risk factors established as causal or likely causal warrant primary prevention efforts, while those classified as probable or possible require further evidence before targeted action can be confidently recommended. Without this explicit causal assessment, the translational strategies discussed in Section 4 would lack an evidence-based foundation. While the preceding sections have separately summarized epidemiological associations and mechanistic evidence, the pivotal question remains: does the totality of evidence support a causal relationship between toxic metal exposure and bladder cancer? To address this, we apply the Bradford Hill criteria—a widely used framework for causal inference from observational data—to the four metals examined in this review (Cox, 2018).

For arsenic, multiple Bradford Hill criteria are convincingly satisfied. The most recent meta-analysis reports a pooled relative risk of 1.60 (95% CI: 1.13–2.27) for arsenic exposure and bladder cancer (Deng et al., 2026), with consistent findings replicated across diverse populations, study designs, and exposure settings (Pal et al., 2020; Chen et al., 2025). The dose-response relationship, while nonlinear, shows a clear increase in risk from 10 to 429 μg/L (Deng et al., 2026), and prospective cohort studies with pre-diagnostic measures establish temporal precedence, even though the long latency period complicates precise exposure-window characterization (Michaud et al., 2004; Zhao et al., 2022). Mechanistically, extensive evidence demonstrates arsenic-induced ROS production, mitochondrial dysfunction, and activation of oncogenic signaling pathways (Ali-El-Dein et al., 2025; Zhou et al., 2021a; Zhou et al., 2021b), providing strong biological plausibility and coherence between epidemiological and experimental observations. We therefore conclude that arsenic exposure is a causal risk factor for bladder cancer.

For cadmium, the evidence is also compelling, though marginally less robust. The pooled RR of 1.38 (95% CI: 1.04–1.82) (Deng et al., 2026), combined with consistent positive associations across tissue, blood, and urine matrices (El-Agrody et al., 2024; Awadalla et al., 2020; Ke et al., 2025), supports a genuine association. Dose-response relationships are suggestive, although formal meta-analyses specific to bladder cancer remain limited (Fanfani et al., 2024), and prospective studies have partially established temporality, though confounding by smoking remains a major interpretive challenge (Kim et al., 2023). Documented oxidative stress, MAPK activation, and miRNA dysregulation support biological plausibility (El-Agrody et al., 2024; Yi et al., 2025; Frin et al., 2024). The main uncertainty is whether cadmium’s effect is independent of smoking or partially mediated by it. We conclude that cadmium is likely a causal risk factor, with the important caveat that its independent contribution requires further elucidation through mediation analyses.

For chromium, the evidence presents a more complex picture. Tissue-based studies consistently show chromium enrichment in tumors and link this accumulation to molecular alterations (Golabek et al., 2017; Sheng et al., 2021), yet serum-based prospective studies have yielded null findings (Caini et al., 2025). This discrepancy likely reflects matrix-specific differences in exposure assessment rather than a genuine absence of effect, but it nevertheless introduces significant uncertainty regarding the systemic exposure levels relevant to bladder carcinogenesis. Mechanistic evidence supporting chromium-induced ROS generation and signaling activation lends biological plausibility (Jomova and Valko, 2011; Nowicka et al., 2020), but inconsistent epidemiological findings across biological matrices preclude a definitive causal determination. We therefore classify chromium as a probable risk factor, acknowledging that confirmatory evidence is needed.

For lead, the evidence is the weakest among the four metals. Cross-sectional studies have shown positive signals, but BMI and nutritional status modify these associations, and prospective data remain sparse (Huang et al., 2025; Vagnoni et al., 2024). The mechanistic evidence is supportive but less robust than for the other metals (Sawicka et al., 2020; Paithankar et al., 2021). We conclude that lead is a possible risk factor, but the evidence remains conditional and insufficient for a definitive causal determination. In summary, the strength of causal evidence follows a clear hierarchy: arsenic (causal) > cadmium (likely causal) > chromium (probable) > lead (possible). This graded framework is reflected in Table 1 and informs the translational and preventive strategies discussed in Section 4. Specifically, arsenic (causal) and cadmium (likely causal) represent the highest-priority targets for environmental regulation, chemoprevention, and biomarker-guided surveillance; chromium (probable) warrants continued mechanistic investigation and improved exposure assessment; and lead (possible) requires further epidemiological clarification before resource-intensive interventions can be justified. This graded approach ensures that the preventive strategies proposed in Section 4 are directly anchored in the strength of causal evidence, rather than being speculative or overly broad.

## 3 Recent advances in research on the core pathogenic mechanisms of toxic metal exposure in the onset and progression of bladder cancer

The mechanistic cascade linking toxic metal exposure to bladder carcinogenesis can be conceptualized as a hierarchical network (Figure 1), beginning with a metal-induced ROS burst and mitochondrial dysfunction, propagating through aberrant activation of oncogenic signaling pathways, and culminating in malignant transformation (Panaiyadiyan et al., 2023; El-Agrody et al., 2024; Ali-El-Dein et al., 2025; Saini et al., 2025; Jomova and Valko, 2011; Nowicka et al., 2020; Baş et al., 2021; Galiniak et al., 2023; Wigner et al., 2021; Gök et al., 2024; Sun Q. et al., 2022; Lewinska et al., 2018; Shah et al., 2025; Bhat et al., 2018). The following subsections systematically dissect each tier of this cascade and extend beyond cancer cell-intrinsic mechanisms to encompass epigenetic alterations, tumor microenvironment remodeling, and metabolic reprogramming, as covered in Sections 3.4 and 3.5.

**FIGURE 1:**
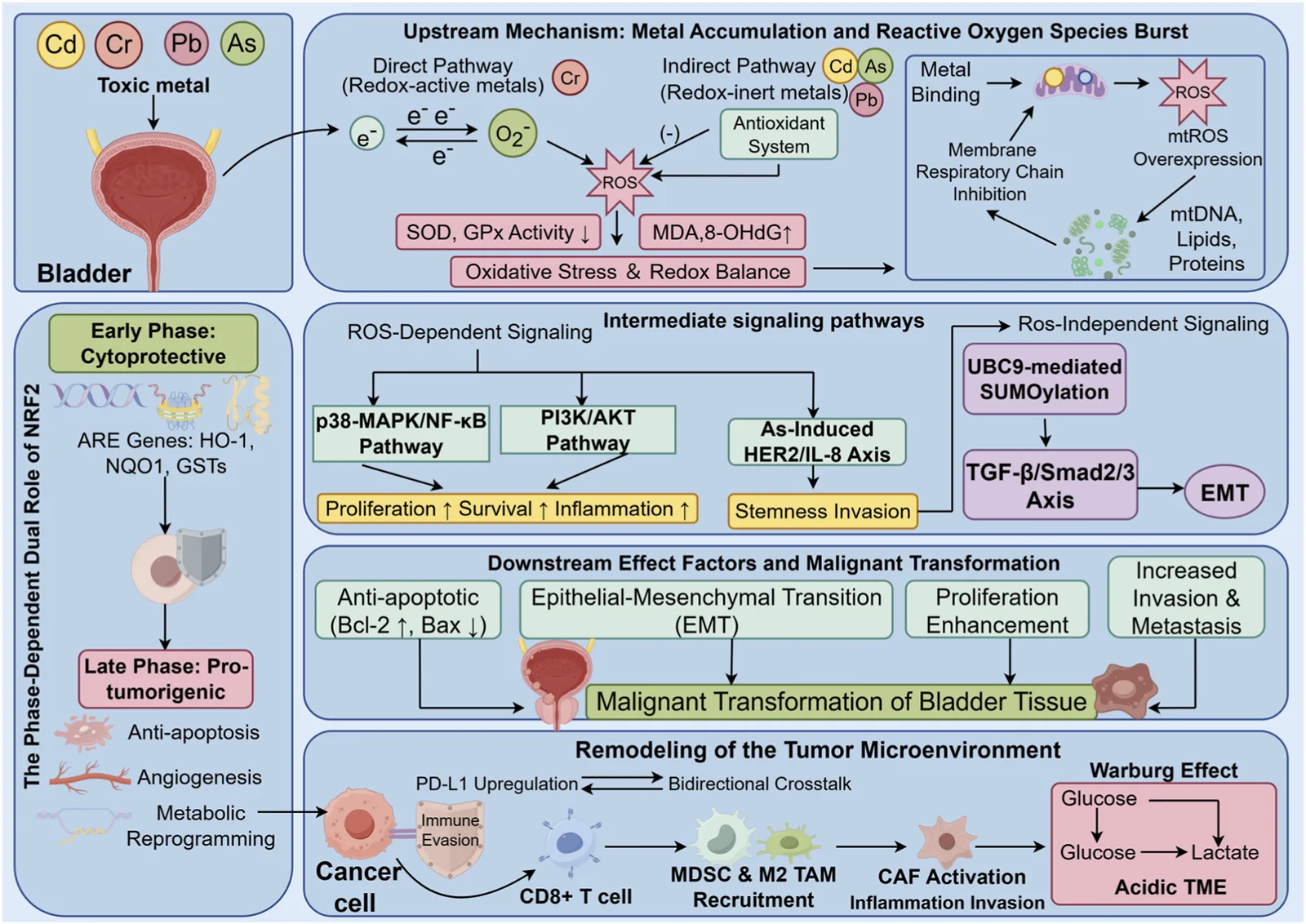
Schematic overview of the mechanisms of toxic metal-driven bladder carcinogenesis. The epidemiological evidence reviewed in Section 2 supports associations between exposure to cadmium, chromium, lead, and arsenic and increased bladder cancer risk. This mechanistic schematic illustrates the molecular circuitry through which these metals may exert their carcinogenic effects. Toxic metals discussed in this review—including cadmium, chromium, lead, and arsenic—accumulate in bladder tissue and trigger excessive reactive oxygen species (ROS) production and mitochondrial dysfunction as upstream initiating events. This upstream damage activates two mechanistically distinct midstream signaling branches. The first branch is ROS-dependent and encompasses the p38-MAPK/NF-κB and PI3K/AKT pathways, which collectively promote cell proliferation, survival, and inflammatory signaling. The second branch is ROS-independent and involves the UBC9-mediated SUMOylation pathway, which enhances TGF-β/Smad2/3 signaling to drive epithelial-mesenchymal transition (EMT). Arsenic additionally engages a unique HER2/IL-8 axis that reinforces stemness and invasive capacity. Nuclear factor E2-related factor 2 (NRF2) exhibits stage-dependent duality, functioning as a cytoprotective antioxidant regulator in early carcinogenesis but acquiring pro-tumorigenic properties in advanced stages through crosstalk with p62 and other oncogenic pathways. These midstream signals converge on downstream cellular outcomes, including EMT, apoptosis resistance, enhanced proliferation, and increased metastatic potential, ultimately leading to malignant transformation and tumor progression. Solid arrows indicate established causal or activating relationships; dashed arrows denote modulatory, stage-dependent, or crosstalk effects. Green boxes indicate potential intervention nodes, including ROS scavengers, mitochondrial protectants, kinase inhibitors targeting MAPK/PI3K/AKT, SUMOylation enzyme inhibitors (UBC9 blockers), and stage-specific NRF2 modulators. This integrated framework consolidates current mechanistic understanding and identifies multiple strategic nodes for therapeutic and preventive intervention. This schematic synthesizes findings from epidemiological, experimental, and preclinical studies. The cited literature supports the causal relationships shown, but not all may be fully established in human bladder cancer.

### 3.1 Upstream events: oxidative stress and mitochondrial damage as the initiating hub

Experimental evidence suggests that toxic metals may contribute to carcinogenesis through two convergent oxidative routes: redox-active metals (e.g., chromium) have been shown to directly catalyze electron transfer to generate ROS, whereas redox-inert metals (e.g., cadmium, lead) appear to indirectly disrupt antioxidant systems, including SOD and GPx, to achieve a similar oxidative burden in cellular and animal models (Jomova and Valko, 2011; Nowicka et al., 2020). This redox imbalance is clinically measurable: bladder cancer patients exhibit significantly elevated serum and urinary oxidative markers—including malondialdehyde (MDA), advanced oxidation protein products (AOPP), total oxidant status (TOS), and oxidative stress index (OSI)—which correlate positively with tumor grade and invasion depth, while tissue superoxide dismutase (SOD) and glutathione peroxidase (GPx) activities are markedly reduced (Panaiyadiyan et al., 2023; Baş et al., 2021; Galiniak et al., 2023; Wigner et al., 2021; Mirkov et al., 2020). Histological studies confirm increased MDA levels and reduced SOD/GPx activity in cancerous tissues (Panaiyadiyan et al., 2023; El-Agrody et al., 2024; Gök et al., 2024). Occupationally, urinary chromium and nickel correlate positively with 8-hydroxy-2′-deoxyguanosine (8-OHdG), a marker of oxidative DNA damage (Su et al., 2019). Animal and cell models also show elevated ROS and depleted GSH after exposure to cadmium, arsenic, mercury, and lead (Mirkov et al., 2020; Song et al., 2019; Yang et al., 2022).

Mitochondria serve as both the primary source and primary target of ROS under toxic metal stress. Significant metal accumulation in bladder cancer tissues directly damages mitochondrial structure and function. This serves as a key event in malignant transformation (Ali-El-Dein et al., 2025). Specifically, toxic metals alter mitochondrial membrane permeability, inhibit respiratory chain complexes, and disrupt electron transport. This uncouples oxidative phosphorylation and impairs ATP synthesis (Sun Q. et al., 2022). *In vitro*, chromium and cadmium significantly reduce mitochondrial membrane potential in T24 bladder cancer cells, indicating compromised structural integrity (Saini et al., 2025). In T24 and RT4 cells, arsenic and cadmium broadly downregulate proteins involved in energy metabolism and mitochondrial homeostasis (Chen et al., 2020). Animal and cellular models show that cadmium induces mitochondrial dysfunction via SIRT1 inhibition. This mechanism is likely relevant to bladder epithelial cells (Wen et al., 2023). As the primary cellular source of ROS, mitochondria are highly susceptible to toxic metal-induced overproduction of mitochondrial ROS (mtROS). This creates a vicious cycle. mtROS further damages mitochondrial DNA, lipids, and proteins. This perpetuates organelle dysfunction and disrupts homeostasis (Sun Q. et al., 2022; Thakur et al., 2021). Clinically, oxidative stress markers are significantly elevated in the serum and tissues of patients with bladder cancer. Total antioxidant status (TAS) and key antioxidant enzyme levels are markedly reduced (Sawicka et al., 2020; Galiniak et al., 2023; Gecit et al., 2017). TIMM44 is highly expressed in bladder cancer tissues. Its inhibition induces mitochondrial depolarisation, ATP depletion, and ROS accumulation. This leads to apoptosis and cell-cycle arrest, further underscoring mitochondrial oxidative stress as a crucial link between metal exposure and bladder cancer development (Zhang et al., 2024).

Importantly, upstream redox imbalance does not operate in isolation; it serves as a potent trigger for a cascade of epigenetic and post-transcriptional alterations. Excessive ROS and mitochondrial damage can directly influence DNA methyltransferase (DNMT) and histone-modifying enzyme activity, while also disrupting the global miRNA network (Lewinska et al., 2018; Shah et al., 2025; Bhat et al., 2018). These changes collectively establish a permissive “molecular switch” that primes the cell for the sustained activation of midstream oncogenic pathways, as detailed in the following section.

### 3.2 Midstream signal transduction: ROS-dependent and -independent activation of oncogenic pathways

Upstream redox imbalance and mitochondrial dysfunction serve as critical triggers for aberrant activation of multiple oncogenic signaling cascades. These midstream signaling events fall into two functionally and mechanistically distinct categories based on ROS dependence. ROS-dependent pathways are directly activated by oxidative stress and include the p38-MAPK and PI3K/AKT axes. ROS-independent pathways operate through alternative mechanisms such as post-translational modifications, exemplified by the SUMOylation-dependent TGF-β/Smad2/3 axis. This distinction has profound implications for both mechanistic understanding and therapeutic targeting. It delineates which signaling nodes are amenable to antioxidant intervention and which require pathway-specific inhibition independent of the redox environment. The following sections systematically dissect this midstream signaling network. They trace the hierarchical flow of information from ROS-triggered events to mechanisms unrelated to oxidative stress. They culminate in the complex crosstalk that amplifies oncogenic output.

The p38-MAPK pathway is among the best-characterized ROS-responsive cascades in toxic metal-associated bladder carcinogenesis. In a study of 125 bladder cancer patients, elevated tissue concentrations of cadmium, cobalt, lead, nickel, and strontium were closely correlated with upregulation of mitochondrial dysfunction-related genes, increased oxidative stress, and robust activation of the p38-MAPK pathway (Ali-El-Dein et al., 2025). This observation has been further corroborated in a cohort of 49 patients with bladder cancer. Chromium and cadmium levels in tumor tissues were positively correlated with increased p38 gene expression (El-Agrody et al., 2024). Mechanistically, reactive oxygen and nitrogen species (RONS) have been implicated in activating p38 MAPK and other classic oncogenic pathways in various cancer models, with downstream effects on proliferation, survival, and migration (Salaheldin et al., 2021). The p38-MAPK pathway does not function in isolation. It serves as an upstream modulator of NF-κB signaling. NF-κB upregulates pro-inflammatory cytokines and reinforces the pro-tumorigenic microenvironment (El-Agrody et al., 2024). Concurrently, p38-MAPK signaling interferes with p21-dependent cell cycle regulation and inhibits p53 tumor-suppressor function. These disruptions undermine growth-control checkpoints while promoting inflammatory cascades (El-Agrody et al., 2024). In parallel, the PI3K/AKT pathway exhibits equally significant ROS-dependent activation in response to toxic metal exposure. AKT is significantly upregulated in bladder cancer tissues. Its expression intensity is positively correlated with chromium and cadmium accumulation (El-Agrody et al., 2024). Reverse validation experiments using ROS-modulating agents have shown that increased ROS levels are associated with inactivation of PI3K/AKT signaling and induction of apoptosis in bladder cancer cells (Kim et al., 2019). Thus, both p38-MAPK and PI3K/AKT operate as proximal ROS sensors. They transduce oxidative stress signals into proliferative and pro-survival outputs. They are the primary conduits through which redox imbalance drives early malignant transformation.

Persistent activation of these pathways is not merely a direct consequence of ROS but is often reinforced by concurrent epigenetic silencing of negative regulators. For instance, hypermethylation of the PTEN promoter, driven by metal-induced DNMT upregulation, can release the brake on PI3K/AKT signaling, thereby amplifying survival signals (Frin et al., 2024; Liu et al., 2020). Similarly, dysregulated miRNAs—such as the upregulation of oncomiR-21 and downregulation of tumor-suppressive miR-200 family members (as summarized in Table 3)—further stabilize this pro-tumorigenic signaling state by targeting key pathway components and EMT regulators (Orang et al., 2025; Yang and Hardy, 2023). Beyond the miRNAs discussed above, miR-155 has also been implicated in metal-associated carcinogenesis. A recent study demonstrated that miR-155 expression is significantly elevated in bladder cancer patients and positively correlates with pro-inflammatory cytokines IL-6 and TNF-α, with concurrent alterations in serum trace element levels (Ay et al., 2025). Additionally, arsenic exposure has been shown to upregulate miR-155 through NF-κB activation, contributing to inflammatory responses and malignant transformation (Chen et al., 2017).

**TABLE 3:** Key miRNAs and epigenetic alterations involved in metal-associated bladder carcinogenesis.

miRNA/Gene	Regulation in metal-exposed cells	Known targets/Function	Effect on signaling/Pathway	Relevant Metal(s)	Refs
miR-21	Upregulated	PTEN, PDCD4	Activates PI3K/AKT, inhibits apoptosis	Cd, As	El-Agrody et al. (2024), Awadalla et al. (2020)
miR-200 family	Downregulated	ZEB1/2, TGF-β pathway components	Promotes EMT, enhances TGF-β/Smad signaling	As, Cd	Orang et al. (2025)
miR-155	Upregulated	SOCS1, FOXO3a	Activates NF-κB, promotes inflammation	As, Cr	Kim et al. (2019), Liu et al. (2020)
PTEN	Hypermethylated	PTEN protein	Removes brake on PI3K/AKT	Cr, Cd	Frin et al. (2024), Liu et al. (2020)
CDKN2A/p16	Hypermethylated	CDKN2A protein	Disrupts cell cycle control	As, Cd	Zhao et al. (2022)
DNMT1, DNMT3b	Upregulated	Global DNA methylation	Promotes hypermethylation of tumor suppressors	Cd, Cr	Saini et al. (2025)

Beyond classical ROS-driven cascades, post-translational modifications have attracted growing attention. SUMOylation operates as a distinct mechanistic route independently of oxidative stress. Elevated serum antimony (Sb) in bladder cancer patients correlates with poor prognosis. Low-dose Sb exposure significantly enhances bladder cancer cell migration and invasion (Yi et al., 2025). Mechanistically, antimony drives SUMO2/3-dependent pan-SUMOylation by activating metal-response elements on the UBC9 promoter, and UBC9 knockdown effectively blocks this SUMOylation and its downstream effects, confirming UBC9’s central regulatory role in this axis (Yi et al., 2025). The ROS-independence of this pathway carries significant therapeutic implications. Antioxidant-based strategies may effectively intercept p38-MAPK and PI3K/AKT activation. However, they would be entirely ineffective against the SUMOylation-TGF-β/Smad2/3 axis. This necessitates developing specific inhibitors of SUMOylation enzymes. This mechanistic divergence underscores the need for combination approaches that simultaneously target both ROS-dependent and ROS-independent signaling compartments to achieve comprehensive inhibition of metal-induced malignant transformation.

Beyond the SUMOylation pathway, arsenic engages a distinct ROS-independent mechanism through the HER2/IL-8 axis. Chronic arsenic exposure induces phosphorylation of human epidermal growth factor receptor 2 (HER2) through coordinated activation of the MAPK, JNK, PI3K/AKT, and GSK3β pathways. Phosphorylated HER2 then drives IL-8 overexpression, which activates ERK, AKT, and STAT3 signaling to promote epithelial-mesenchymal transition (EMT) and upregulate the cancer stem cell (CSC) marker CD44 in human uroepithelial cells (Zhou et al., 2021a; Zhou et al., 2021b). This HER2/IL-8 axis thus serves as a critical mechanistic link between arsenic exposure and the acquisition of aggressive, stemness-associated phenotypes. Notably, genistein has been shown to inhibit arsenic-induced HER2 phosphorylation and to downregulate its downstream signaling cascades, thereby blocking EMT progression and reducing CD44 expression, offering a potential chemopreventive strategy against arsenic-induced bladder cancer (Zhou et al., 2021a).

Nuclear factor E2-related factor 2 (NRF2) exemplifies the contextual complexity of redox signaling. In normal urothelial cells and in early premalignant stages, NRF2 activation upregulates antioxidant response element (ARE)-driven genes (e.g., HO-1, NQO1, GSTs), providing cytoprotection against metal-induced oxidative damage (Salaheldin et al., 2021; Zhang and Costa, 2021). However, in established tumors, NRF2 is frequently overexpressed or constitutively activated, promoting anti-apoptosis, angiogenesis, metabolic reprogramming, and chemoresistance (Xu et al., 2017). This stage-dependent duality—protective in early carcinogenesis but pro-tumorigenic in advanced disease—is not merely a paradox but a critical mechanistic feature that complicates therapeutic targeting. Furthermore, NRF2 engages in extensive crosstalk with the MAPK, PI3K/AKT, and SUMOylation pathways, forming a complex signaling network in which activation of one node can positively or negatively modulate others (El-Agrody et al., 2024; Zhang and Costa, 2021). For instance, p38-MAPK can both directly promote proliferation and indirectly enhance NF-κB-mediated inflammation, thereby modulating NRF2 activity through redox-dependent feedback loops. The SUMOylation-TGF-β/Smad2/3 axis can synergize with PI3K/AKT to drive EMT, while PI3K/AKT-mediated survival signals may counteract ROS-induced apoptosis (Yi et al., 2025). This dynamic interplay, not the linear sum of individual pathway activities, produces the net oncogenic output.

A major limitation of current mechanistic models is that most evidence comes from *in vitro* cell lines exposed to acute, high-dose metal concentrations that often far exceed human physiological or environmental levels (Panaiyadiyan et al., 2023; El-Agrody et al., 2024; Rej et al., 2026). These experimental conditions may not recapitulate the chronic, low-dose, multi-metal co-exposure scenarios typical of real-world settings. Moreover, using immortalized cancer cell lines—rather than primary urothelial cells or patient-derived organoids—may skew signaling responses and obscure tissue-specific effects. Therefore, while the proposed hierarchical model is mechanistically coherent, its direct translatability to human bladder carcinogenesis requires rigorous validation in physiologically relevant models and, ideally, in prospective cohorts with matched biospecimens. Emerging technologies such as single-cell sequencing and post-translational modification (PTM) proteomics offer unprecedented opportunities to dissect these pathways in clinical samples at spatiotemporal resolution, which will be essential for confirming functional causality. Table 4 summarizes the key signaling pathways discussed above.

**TABLE 4:** Key signaling pathways involved in toxic metal-driven bladder carcinogenesis.

Pathway	ROS dependence	Activating metals	Molecular events	Functional outcomes	Evidence source
p38-MAPK/NF-κB	ROS-dependent	Cd, Cr, Pb, As, Co	p38 phosphorylation; NF-κB activation; inflammatory cytokine upregulation	Proliferation, survival, inflammation; disrupts p53/p21 checkpoints	Tissue studies (El-Agrody et al., 2024; Ali-El-Dein et al., 2025); RONS activation (Salaheldin et al., 2021)
PI3K/AKT	ROS-dependent	Cd, Cr	AKT upregulation; survival signal activation	Cell survival; apoptosis resistance	AKT correlates with metal levels (El-Agrody et al., 2024); ROS inhibition induces apoptosis (Wen et al., 2023)
SUMOylation/TGF-β/Smad2/3	ROS-independent	Sb (antimony)	UBC9-mediated SUMO2/3 modification; Smad2/3 phosphorylation; EMT induction	Migration, invasion, metastasis	Antimony exposure model (Xu et al., 2017); UBC9 knockdown blocks effects (Xu et al., 2017)
NRF2/ARE	Stage-dependent	Cd, Cr, As, Pb	ARE-driven gene activation (HO-1, NQO1, GSTs)	Early: cytoprotection; Late: chemoresistance, metabolic reprogramming, angiogenesis	Stage-dependent duality (Xu et al., 2017; Zhang and Costa, 2021); p62 crosstalk (Zhang and Costa, 2021)
HER2/IL-8	ROS-independent (Arsenic-specific)	As	HER2 phosphorylation via MAPK/JNK/PI3K/AKT/GSK3β; IL-8 overexpression; ERK/AKT/STAT3 activation	EMT induction; CD44 upregulation; acquisition of cancer stem cell properties	Arsenic-exposed uroepithelial cell models (Zhou et al., 2021a; Zhou et al., 2021b); genistein blocks HER2 phosphorylation and downstream effects (Zhou et al., 2021a)

### 3.3 Downstream cellular outcomes: EMT, apoptosis resistance, and malignant transformation

The convergence of upstream oxidative injury and midstream signaling aberrations ultimately translates into profound changes in cellular behavior. Midstream networks do not operate in parallel; they converge on a core set of transcription factors that govern epithelial-mesenchymal transition (EMT). NF-κB-induced inflammatory signals synergize with TGF-β/Smad signaling to repress E-cadherin, while PI3K/AKT-mediated survival signals stabilize mesenchymal markers. This transcriptional reprogramming, combined with a disrupted apoptotic balance, collectively drives the shift from normal urothelial homeostasis towards unchecked proliferation, apoptotic resistance, and invasive potential. Beyond energy metabolism and oxidative damage, mitochondria also regulate endogenous apoptotic pathways.

Their apoptotic balance is significantly disrupted by toxic metal exposure. In bladder cancer tissues, the anti-apoptotic protein B-cell lymphoma 2 (Bcl-2) is overexpressed, while the pro-apoptotic protein BCL2-associated X protein (Bax) is downregulated. This imbalance correlates positively with chromium and cadmium levels (El-Agrody et al., 2024; Sawicka et al., 2020). These findings suggest that metal accumulation may disrupt the balance between pro- and anti-apoptotic signals, potentially inhibiting normal apoptotic repair mechanisms (El-Agrody et al., 2024; Isermann et al., 2017). This creates a favorable microenvironment for malignant transformation and tumor survival (Sawicka et al., 2020). The SUMOylation pathway plays a particularly critical role in driving metastatic phenotypes. Antimony-induced SUMO2/3-dependent pan-SUMOylation enhances Smad2/3 phosphorylation, inducing EMT and promoting tumor invasion (Yi et al., 2025). UBC9 knockdown effectively blocks both SUMOylation and EMT, validating this pathway as a central driver of toxic metal-induced metastasis. MAPK and PI3K/AKT synergistically promote proliferation and survival. The SUMOylation-TGF-β/Smad2/3 axis drives EMT and invasion. NF-κB-mediated upregulation of pro-inflammatory cytokines reinforces the pro-tumorigenic microenvironment. Critically, crosstalk among these pathways amplifies oncogenic output. NRF2’s stage-dependent switch from protective to pro-tumorigenic further complicates the landscape. The net effect is a coordinated shift from normal urothelial homeostasis to malignant transformation. This is characterized by unchecked proliferation, apoptotic resistance, and invasive potential. The causal chain from toxic metal exposure to bladder cancer progression follows a clear trajectory. Upstream, ROS overproduction and mitochondrial dysfunction create a pro-oxidant, pro-inflammatory microenvironment. Midstream, aberrant activation of MAPK, PI3K/AKT, and SUMOylation-TGF-β/Smad2/3 pathways transduce these stress signals into sustained proliferative and survival signals. Downstream, disruption of apoptotic balance, EMT induction, and enhanced invasive capacity culminate in malignant transformation and tumor progression. This integrated framework consolidates current mechanistic understanding. It also identifies multiple nodes—ROS, mitochondria, specific kinases, and SUMOylation enzymes—as potential targets for therapeutic and preventive intervention.

### 3.4 Impact on the tumor microenvironment: cellular, immune, and metabolic interactions

Beyond their direct effects on urothelial cells, toxic metals exert profound indirect effects on the bladder tumor microenvironment (TME), the complex ecosystem comprising cancer cells, stromal cells, immune infiltrates, and metabolic intermediates that collectively shape tumor progression and therapeutic response (Hatogai and Sweis, 2020; Patwardhan and Mahendran, 2023). Although less extensively studied than the intracellular pathways described above, emerging evidence indicates that metal-induced oxidative stress and signaling aberrations extend beyond cancer cells, reprogramming the TME in ways that favor malignant progression.

Toxic metals can subvert antitumor immunity through multiple mechanisms. Arsenic exposure has been shown to promote an immunosuppressive milieu by regulating PD-L1 expression on bladder cancer cells via STAT3 and NF-κB activation, thereby facilitating immune evasion (Liu et al., 2018; Almeida Rodrigues et al., 2023; Cai et al., 2021). Concurrently, metals may impair tumor-infiltrating lymphocyte (TIL) and natural killer (NK) cell function through ROS-mediated oxidative damage, while promoting the recruitment of immunosuppressive myeloid-derived suppressor cells (MDSCs) and tumor-associated macrophages (TAMs) with an M2-like phenotype (Miyake et al., 2016; Singhal et al., 2022; Kennel and Greten, 2021; Sun Y. et al., 2022). These collective changes tilt the local immune balance from tumor surveillance toward immune tolerance.

Metal-induced chronic inflammation, mediated through NF-κB and IL-6/STAT3 signaling, creates a pro-tumorigenic inflammatory microenvironment characterized by sustained production of cytokines and chemokines that promote angiogenesis, tissue remodeling, and invasion. Cancer-associated fibroblasts (CAFs), when exposed to metal stress, acquire an activated phenotype that secretes extracellular matrix-degrading enzymes and growth factors, facilitating tumor cell dissemination. In high-arsenic areas, elevated IL-8 secretion may further recruit neutrophils and promote an inflammatory niche conducive to metastasis.

Toxic metals profoundly alter cellular metabolism within the TME. Arsenic and cadmium disrupt mitochondrial oxidative phosphorylation, thereby forcing a metabolic shift toward aerobic glycolysis (the Warburg effect) to sustain ATP production in cancer cells, even in the presence of oxygen (Yu et al., 2025; Tarhonska et al., 2023; Ding et al., 2023). This metabolic reprogramming not only fuels cancer cell proliferation but also generates a lactate-rich, acidified TME that suppresses effector immune cell function while favoring the expansion of immunosuppressive populations (Yu et al., 2025; Christiansen et al., 2026; Oluwatola et al., 2026). Additionally, metal-induced alterations in lipid metabolism and amino acid homeostasis may further shape the metabolic landscape, influencing both cancer cell survival and immune cell fitness (Zhao et al., 2021; González-Al et al., 2026; Li et al., 2023).

Critically, these TME alterations do not operate in isolation but engage in bidirectional crosstalk with the cancer cell-intrinsic signaling pathways described in Sections 3.1–3.3. For instance, metal-induced ROS production in cancer cells can be amplified by paracrine signals from CAFs and inflammatory cells, creating a feed-forward loop that sustains oxidative stress and activates signaling pathways. Conversely, cancer cell-derived exosomes containing metal-induced molecular cargo may educate stromal cells to adopt pro-tumorigenic phenotypes, propagating the carcinogenic influence beyond the initially exposed cells. Understanding these complex TME interactions is essential for developing comprehensive therapeutic strategies that target not only cancer cells but also the supportive microenvironment that enables their survival and progression.

Tumor Heterogeneity Modulates the Mechanistic Cascade Illustrated in Figure 1. While Figure 1 presents a unified mechanistic cascade from metal exposure to malignant transformation, this cascade operates within a spatially and temporally heterogeneous tumor microenvironment. The TME is characterized by gradients of oxygen tension, nutrient availability, pH, and immune infiltration, all of which can profoundly modulate the activation thresholds and output of the signaling pathways depicted in Figure 1. For instance, hypoxia—a common feature of the tumor interior—can synergize with metal-induced ROS to amplify HIF-1α and NF-κB signaling in central tumor regions, while perivascular zones with higher oxygen tension may exhibit preferential PI3K/AKT activation driven by growth factor gradients (Castelli et al., 2021; Akinsulie et al., 2023; Yeh et al., 2018). Similarly, the ROS-dependent p38-MAPK/NF-κB pathway may be constitutively activated in inflammatory niches enriched with tumor-associated macrophages and cytokines, whereas the ROS-independent SUMOylation/TGF-β/Smad2/3 axis may dominate in stromal-rich regions where TGF-β is abundantly secreted by cancer-associated fibroblasts (Barata and Dick, 2020; Yee et al., 2021; Chang and Ding, 2018). This spatial heterogeneity implies that the same metal exposure can elicit divergent molecular responses across different tumor subregions, potentially driving clonal evolution and therapeutic resistance.

Furthermore, inter-patient heterogeneity—driven by differences in genetic background, smoking status, nutritional status, and co-exposure to other environmental toxicants—adds another layer of complexity. Polymorphisms in genes governing antioxidant defense (e.g., SOD2, GPX1) or metal metabolism (e.g., AS3MT, GST) can modulate individual susceptibility to metal-induced pathway activation, resulting in distinct molecular phenotypes even under comparable exposure levels (Chen et al., 2018; Huang et al., 2018; Zhou et al., 2018; de la Rosa et al., 2017). This heterogeneity has important clinical implications: therapeutic strategies targeting a single pathway may be effective only in tumor regions where that pathway is dominantly activated, while other regions may escape through alternative signaling branches. Thus, the hierarchical framework proposed in Figure 1 should be viewed not as a rigid, linear sequence but as a flexible, context-dependent network whose output is continuously shaped by the tumor microenvironment and host factors. Future research integrating spatial transcriptomics and single-cell profiling will be essential to map the heterogeneity of metal-induced pathway activation across tumor subregions and to guide the development of combination therapies that account for this complexity.

### 3.5 Integrated mechanistic framework: a hierarchical and interconnected network

Synthesizing the evidence above, we propose a hierarchical, interconnected framework for toxic metal-driven bladder carcinogenesis that integrates the discussed molecular events into a cohesive model. This framework can be conceptualized as a five-tiered cascade. Metal accumulation (Cd, Cr, Pb, As) triggers immediate cellular stress responses, including the induction of metallothionein (MT) for metal detoxification and the activation of the redox-sensitive transcription factor NRF2 (Frin et al., 2024; Greco et al., 2019; Wang et al., 2025). NRF2’s function is paradoxical at this stage: it provides cytoprotection through antioxidant gene expression in normal cells, but sustained activation in chronically exposed cells can promote survival and metabolic reprogramming (Qi et al., 2023; Truong et al., 2025). The oxidative stress and mitochondrial dysfunction initiated in Tier 1 drive profound epigenetic alterations and global miRNA dysregulation. For example, ROS-induced DNMT1 upregulation silences genes like PTEN and CDKN2A, while oncomiRs (e.g., miR-21, miR-155) are induced, and tumor-suppressive miRs (e.g., miR-200 family, miR-145) are repressed (Frin et al., 2024; Lewinska et al., 2018; Wang and Men, 2015). This epigenetic and miRNA network acts as a “molecular memory” that amplifies and perpetuates initial stress signals, establishing a pro-carcinogenic transcriptional landscape (Lewinska et al., 2018; Qi et al., 2023; Truong et al., 2025).

The rewired epigenetic and miRNA landscape creates a permissive environment for the perpetual activation of core oncogenic pathways. PTEN silencing unleashes PI3K/AKT (Frin et al., 2024). NF-κB is constitutively activated by miR-21-mediated suppression of its inhibitor, and TGF-β signaling is enhanced by the loss of miR-200 family members that normally target TGF-β pathway components. These pathways become self-sustaining and no longer depend solely on ongoing ROS production (Qi et al., 2023; Truong et al., 2025). Sustained midstream signaling converges on key transcription factors to drive EMT while disrupting the apoptotic balance (Bcl-2/Bax) and promoting proliferation and invasion. This represents the final common path leading to malignant transformation.

The malignant cells, equipped with EMT and survival traits, actively remodel their TME through secreted factors (e.g., IL-6, IL-8, TGF-β), promoting CAF activation, immunosuppressive cell recruitment, and metabolic reprogramming as detailed in Section 3.4. In turn, the hostile TME provides paracrine signals that further fuel Tiers 2–4, creating a vicious cycle of tumor progression. This integrated model highlights the multi-layered nature of metal carcinogenesis, underscoring that effective prevention and therapy must consider this interconnected web of molecular events rather than targeting single pathways in isolation (Figure 2).

**FIGURE 2:**
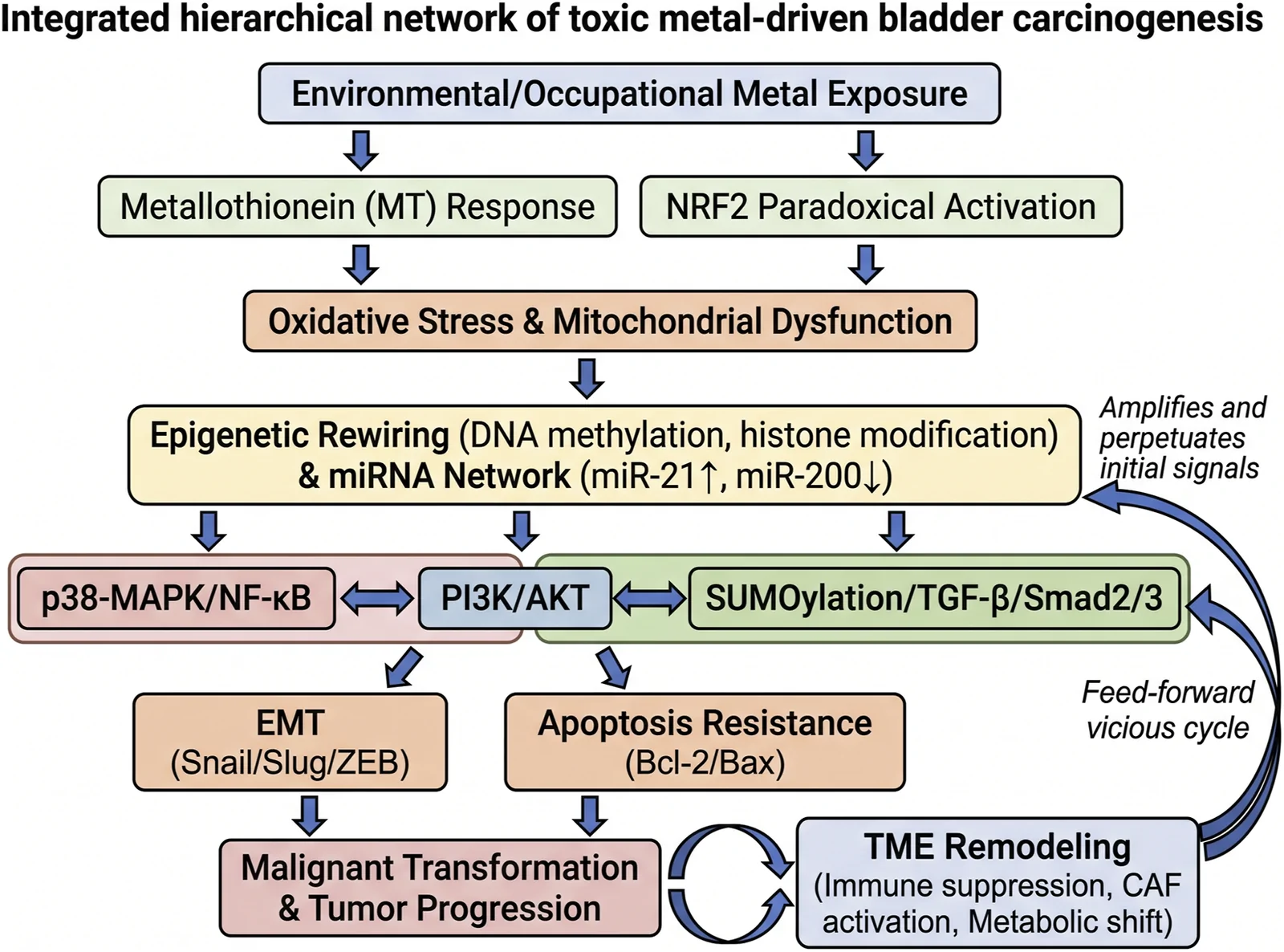
Integrated hierarchical network of toxic metal-driven bladder carcinogenesis. This schematic synthesizes the hierarchical framework proposed in Section 3.5 and illustrates the cascade from metal exposure to malignant transformation. The framework comprises five interconnected tiers. **Tier 1**: Metal exposure (Cd, Cr, Pb, As) triggers MT induction and NRF2 paradoxical activation, which is cytoprotective in early stages but pro-tumorigenic in advanced disease. **Tier 2**: Oxidative stress drives epigenetic rewiring (DNA methylation, histone modifications) and miRNA dysregulation (miR-21↑, miR-200↓), establishing a pro-carcinogenic transcriptional landscape. **Tier 3**: These changes enable sustained activation of three midstream signaling modules—p38-MAPK/NF-κB, PI3K/AKT, and SUMOylation/TGF-β/Smad2/3—with extensive crosstalk among them. **Tier 4**: Converging signals activate EMT transcription factors (Snail, Slug, ZEB) and disrupt apoptotic balance (Bcl-2/Bax), driving malignant transformation. **Tier 5**: Malignant cells remodel the TME through immune suppression, CAF activation, and metabolic shifts, which in turn feedback to reinforce upstream tiers. Solid arrows indicate activation; dashed arrows denote modulatory or crosstalk effects; curved arrows represent feed-forward loops. The curved arrows emphasize that TME remodeling not only results from upstream molecular alterations but also reinforces them, forming a self-perpetuating vicious cycle that drives tumor progression.

## 4 Translational implications and integrated prevention strategies

The mechanistic framework described above provides a rich landscape for translational applications, ranging from biomarker development to targeted interventions and environmental control. The causal hierarchy established in Section 2.3—arsenic (causal), cadmium (likely causal), chromium (probable), and lead (possible)—provides the foundational rationale for the intervention strategies outlined below. This graded framework ensures that prevention efforts are proportionate to the strength of evidence: the strongest causal evidence justifies the most assertive environmental and clinical actions. However, translating these findings into clinical and public health practice faces significant bottlenecks. In this section, we critically evaluate the translational potential of various strategies, prioritize them by their current readiness, and explicitly address the barriers that must be overcome. The mechanistic framework described above provides a rich landscape for translational applications, ranging from biomarker development to targeted interventions and environmental control. However, translating these findings into clinical and public health practice faces significant bottlenecks. In this section, we critically evaluate the translational potential of various strategies, prioritize them by their current readiness, and explicitly address the barriers that must be overcome. The causal hierarchy established in Section 2.3—arsenic (causal), cadmium (likely causal), chromium (probable), and lead (possible)—provides the foundational rationale for the intervention strategies outlined below. This graded framework ensures that prevention efforts are proportionate to the strength of evidence: the strongest causal evidence justifies the most assertive environmental and clinical actions.

### 4.1 Biomarkers: from exposure to effect to susceptibility

Mechanistic research has progressively identified biomarkers spanning the continuum from exposure assessment to disease prediction, logically arrayed along the causal chain linking environmental insult to clinical outcome. Along this continuum, exposure biomarkers—quantifiable metal concentrations in blood, urine, tissue, or nails—directly reflect the internal dose of toxicants accumulated in the body; effect biomarkers, encompassing oxidative stress indicators such as MDA, 8-OHdG, AOPP, and glutathione imbalance, capture the early biological consequences of metal-induced redox disruption; while susceptibility biomarkers, including genetic polymorphisms in genes governing metal metabolism and antioxidant defense such as GST, SOD2, GPX1, and AS3MT, further modulate inter-individual vulnerability by influencing detoxification capacity and oxidative stress resilience (Panaiyadiyan et al., 2023; Nowicka et al., 2020; Gök et al., 2024; Chen et al., 2018; Huang et al., 2018). Multiple studies consistently show that patients with bladder cancer have elevated oxidative stress markers (e.g., AOPP, TOS, OSI, MDA) compared with healthy controls, and these markers correlate positively with tumor stage and grade (Nowicka et al., 2020; Mirkov et al., 2020). At the tissue level, elevated concentrations of lead, chromium, and cadmium in cancerous tissues are accompanied by increased MDA and decreased SOD activity (Chung et al., 2017). In high-Arsenic areas, recurrent tumors contain extremely high arsenic levels, which correlate with aggressive progression (Pal et al., 2020). Emerging evidence supports the prognostic value of metal levels: elevated blood cadmium levels in MIBC patients positively correlate with miRNA-21 expression, suggesting a link between metal burden and an aggressive phenotype (El-Agrody et al., 2024).

Inter-individual susceptibility is another critical dimension. Polymorphisms in GST genes (e.g., GSTM1 null genotype) have been associated with increased bladder cancer risk, potentially modified by smoking (Chen et al., 2018; Zhou et al., 2018). Variations in AS3MT may modulate arsenic-related bladder cancer risk (Huang et al., 2018; de la Rosa et al., 2017). Similarly, SOD2 (rs4880) and GPX1 (Pro198Leu) polymorphisms have been linked to susceptibility (Zhang et al., 2020; Wang et al., 2017), although findings are not entirely consistent (Hadami et al., 2016; Nikic et al., 2018). Integrating toxic metal burden testing, oxidative stress quantification, and susceptibility genotyping could establish a multidimensional biomarker panel that captures current exposure, early biological effects, and genetic predisposition, thereby enhancing early detection, risk stratification, and personalized surveillance. The proposed multi-tiered biomarker panel is summarized in Table 5.

**TABLE 5:** Multi-tiered biomarker panel for toxic metal-associated bladder cancer.

Tier	Biomarker type	Examples	Clinical application	Evidence
Exposure	Metal concentrations	Blood/urine/tissue/nail levels of Cd, Cr, Pb, As	Assess current/internal dose; identify high-exposure individuals	Tissue levels consistently elevated in cancer patients (Panaiyadiyan et al., 2023; Golabek et al., 2017; Abdel-Gawad et al., 2016); nails reflect chronic exposure (Salcedo-Bellido et al., 2021)
Effect	Oxidative stress markers	MDA, 8-OHdG, AOPP, TOS, OSI, GSH/GSSG ratio	Detect early biological damage; monitor disease progression	Elevated in cancer patients; correlate with stage/grade (Galiniak et al., 2023; Wigner et al., 2021)
Susceptibility	Genetic polymorphisms	GSTM1 null, SOD2 rs4880, GPX1 Pro198Leu, AS3MT variants	Identify genetically vulnerable subgroups; personalize surveillance	GSTM1 null associated with increased risk (Barata and Dick, 2020; Chang and Ding, 2018); SOD2 and GPX1 linked to susceptibility (Zhang et al., 2020; Wang et al., 2017)

### 4.2 Intervention strategies: from mechanistic rationale to clinical application

The mechanistic framework described in Section 3 provides a biological rationale for multiple therapeutic strategies. Still, the translational landscape spans a broad spectrum—from preclinical candidate compounds with limited evidence to FDA-approved agents with established clinical efficacy in bladder cancer. In this section, we critically evaluate intervention strategies across this spectrum, prioritizing those with the strongest clinical evidence while acknowledging the mechanistic promise and current limitations of emerging approaches.

#### 4.2.1 FDA-approved agents with established clinical efficacy

Several agents have received FDA approval for bladder cancer and directly or indirectly intersect with the mechanistic pathways discussed in this review. Their inclusion is essential for a clinically relevant translational discussion.

Enfortumab Vedotin is an ADC targeting NECTIN-4. It has demonstrated significant overall survival benefit in patients with locally advanced or metastatic urothelial carcinoma who have previously received platinum-based chemotherapy and PD-1/PD-L1 inhibitors, leading to FDA approval as a single agent and in combination with pembrolizumab (Rosenberg et al., 2019; Taguchi et al., 2023; Powles et al., 2021; Brave et al., 2024). Disitamab Vedotin (RC48) is a HER2-targeting ADC that, while not yet FDA-approved, has shown promising efficacy in HER2-positive locally advanced or metastatic urothelial carcinoma in clinical trials conducted in China (Chen et al., 2023; Sheng et al., 2024). Arsenic exposure has been shown to upregulate HER2 signaling in bladder cancer cells, suggesting a potential mechanistic rationale for combining HER2-targeted therapies with strategies that modulate metal-induced signaling aberrations—an area that warrants dedicated investigation (Du et al., 2021; Fang et al., 2025). The NRF2 pathway may also contribute to ADC resistance, as NRF2-mediated upregulation of antioxidant and detoxification genes could limit the efficacy of cytotoxic payloads (Semeniuk-Wojtaś et al., 2023; Luo et al., 2022).

Pembrolizumab, a PD-1 inhibitor, has received FDA approval for subsets of patients with bladder cancer, including those with locally advanced or metastatic urothelial carcinoma who have progressed on or after platinum-containing chemotherapy (Bellmunt et al., 2017), as well as for cisplatin-ineligible patients in the first-line setting whose tumors express PD-L1 (Balar et al., 2017). Pembrolizumab in combination with enfortumab vedotin has been approved as perioperative treatment for cisplatin-ineligible patients with muscle-invasive bladder cancer (Lawrence, 2026). Notably, toxic metal exposure has been shown to upregulate PD-L1 expression in lung epithelial cells and modulate inflammatory responses in murine models, suggesting a potential link between metal exposure and immune checkpoint regulation that warrants investigation in bladder cancer (Xu et al., 2024; Wang et al., 2024; Voli et al., 2020; Das et al., 2023). Whether this mechanism is operative in metal-exposed bladder cancer patients and whether immune checkpoint blockade would be particularly beneficial in this population remain open questions.

#### 4.2.2 Preclinical and early-stage candidate interventions

Beyond approved therapies, several experimental strategies have shown mechanistic promise but currently lack clinical validation. Agents such as lycopene, phytosterols, kaempferol, and excavatolide C have demonstrated antioxidant and signal-modulating activities *in vitro* and animal models, primarily through ROS scavenging, mitochondrial protection, and modulation of the NF-κB, PI3K/AKT, and MAPK pathways (Yang et al., 2022; Kim et al., 2019; Chen et al., 2018; Wang et al., 2022). Most are dietary components with established safety profiles, making them attractive for primary prevention. However, their poor bioavailability and low target-tissue distribution are major translational hurdles, and no clinical evidence yet supports their efficacy specifically in patients with bladder cancer. Rigorous pharmacokinetic optimization—potentially through advanced delivery systems such as liposomes or polymer nanoparticles—and well-designed prevention trials in high-risk populations are prerequisites for clinical translation.

The demethylating agent decitabine has shown preclinical activity in cadmium- and chromium-exposed bladder cancer cells, inhibiting proliferation and downregulating DNMT1 and DNMT3b. It has entered Phase I trials for recurrent bladder cancer (Saini et al., 2025). However, its non-specific systemic demethylation and bone marrow suppression pose significant clinical risks, requiring dose optimization or intravesical delivery. Targeting UBC9 to block antimony-induced SUMO2/3-dependent TGF-β/Smad2/3 activation and EMT represents a mechanistically novel anti-metastatic strategy, but current evidence is limited to proof-of-concept cellular data (Yi et al., 2025). Lead compound optimization and rigorous toxicological evaluation are needed before considering clinical development.

As discussed in Section 3.2, NRF2’s stage-dependent duality presents a therapeutic paradox. This duality requires precise patient stratification: NRF2 activators may be considered for high-risk populations with chronic low-level exposure, while NRF2 inhibitors could be explored for advanced tumors with NRF2 hyperactivation (Xu et al., 2017; Emami et al., 2022). However, both approaches remain investigational and await the development and validation of reliable companion diagnostics.

#### 4.2.3 An integrated framework for clinical translation

The agents discussed above span a continuum from established clinical therapies—including enfortumab vedotin and pembrolizumab—to promising candidates such as disitamab vedotin, and experimental strategies with mechanistic promise such as natural antioxidants, decitabine, and SUMOylation inhibitors. Translating these mechanistic insights into measurable clinical benefit requires systematic progress along several interconnected research trajectories.

First, researchers must discover and validate predictive biomarkers to identify which metal-exposed patients are most likely to respond to specific agents, enabling precise patient stratification for clinical trials. Second, pharmacokinetic optimization is urgently needed to overcome the bioavailability barriers of natural compounds, potentially through advanced delivery systems such as liposomes or polymer nanoparticles. Third, rational combination strategies warrant exploration, for instance, pairing HER2-targeted ADCs with agents that modulate metal-induced HER2 upregulation. Fourth, large-scale randomized controlled trials of lifestyle interventions and antioxidant supplementation should be conducted specifically in populations with documented metal exposure. Once addressed, these research priorities will provide the evidence base for the multi-tiered prevention framework discussed in Section 4.4, which operationalizes these strategies in clinical and public health practice.

### 4.3 Environmental control and high-risk population surveillance

Reducing environmental pollution from cadmium, lead, chromium, and arsenic is a fundamental but underutilized primary prevention measure. Mining activities, industrial runoff, and soil contamination continuously expose populations through the water and food chains (Yao et al., 2021; Qiao et al., 2023). Technologies such as multi-source mineral composite calcined materials (MMCCM) have shown promise in suppressing metal release from sediments (Yao et al., 2024), and soil remediation efforts have been linked to reduced ecological risks (Yang et al., 2025). However, current environmental policies remain broadly focused on public health protection and have not been systematically integrated into urological cancer prevention systems. Future efforts must bridge this gap by establishing exposure surveillance in high-risk regions and leveraging biomonitoring to identify vulnerable subgroups (Isermann et al., 2017). The limitations of current biomonitoring—such as the poor correlation between serum chromium and tissue accumulation—highlight the need for more targeted biomarker panels that combine exposure, effect, and susceptibility markers to guide screening and early intervention.

### 4.4 Integrated multi-tiered prevention framework

Building upon the biomarker framework outlined in Section 4.1 and the translational research priorities identified in Section 4.2.3, we propose an integrated prevention system that operates across three interconnected, progressively targeted levels, with biomarker profiling serving as the common thread guiding decision-making at each stage. At the foundational level, primary prevention aims to reduce population-level exposure through environmental regulation and lifestyle counseling in metal-exposed communities, thereby addressing the root cause before any biological damage occurs. At the secondary level, biomarker-based screening identifies high-risk individuals with confirmed exposure who may benefit from chemopreventive interventions, such as antioxidant supplementation or stage-appropriate NRF2 modulation, to intercept or reverse early carcinogenic changes. At the tertiary level, molecular profiling informs the deployment of targeted therapies, including decitabine for epigenetic reprogramming and SUMOylation inhibitors for metastatic disease, to manage established tumors and prevent further progression in affected patients (Yi et al., 2025; Xu et al., 2017; Saini et al., 2025). This framework requires interdisciplinary collaboration among environmental health scientists, analytical toxicologists, molecular epidemiologists, and urologic oncologists. Rigorous validation through prospective cohort studies and randomized controlled trials, specifically designed for metal-exposed populations, is essential to translate this framework into measurable public health benefits. The graded causal framework established in Section 2.3 (arsenic > cadmium > chromium > lead) provides a rational basis for prioritizing prevention resources: high-risk populations in arsenic-endemic regions should be prioritized for surveillance and intervention, followed by cadmium-exposed occupational cohorts, while chromium and lead require further evidence before targeted public health action can be confidently recommended.

## 5 Limitations and future perspectives

The preceding sections have synthesized a substantial body of evidence linking toxic metal exposure to bladder cancer through a coherent mechanistic framework. Despite this progress, several critical knowledge gaps persist, limiting our understanding and impeding translation into clinical and public health practice. In this section, we identify these limitations and discuss promising research trajectories that may help address them, while critically acknowledging the practical challenges inherent in each proposed direction (Figure 3).

**FIGURE 3:**
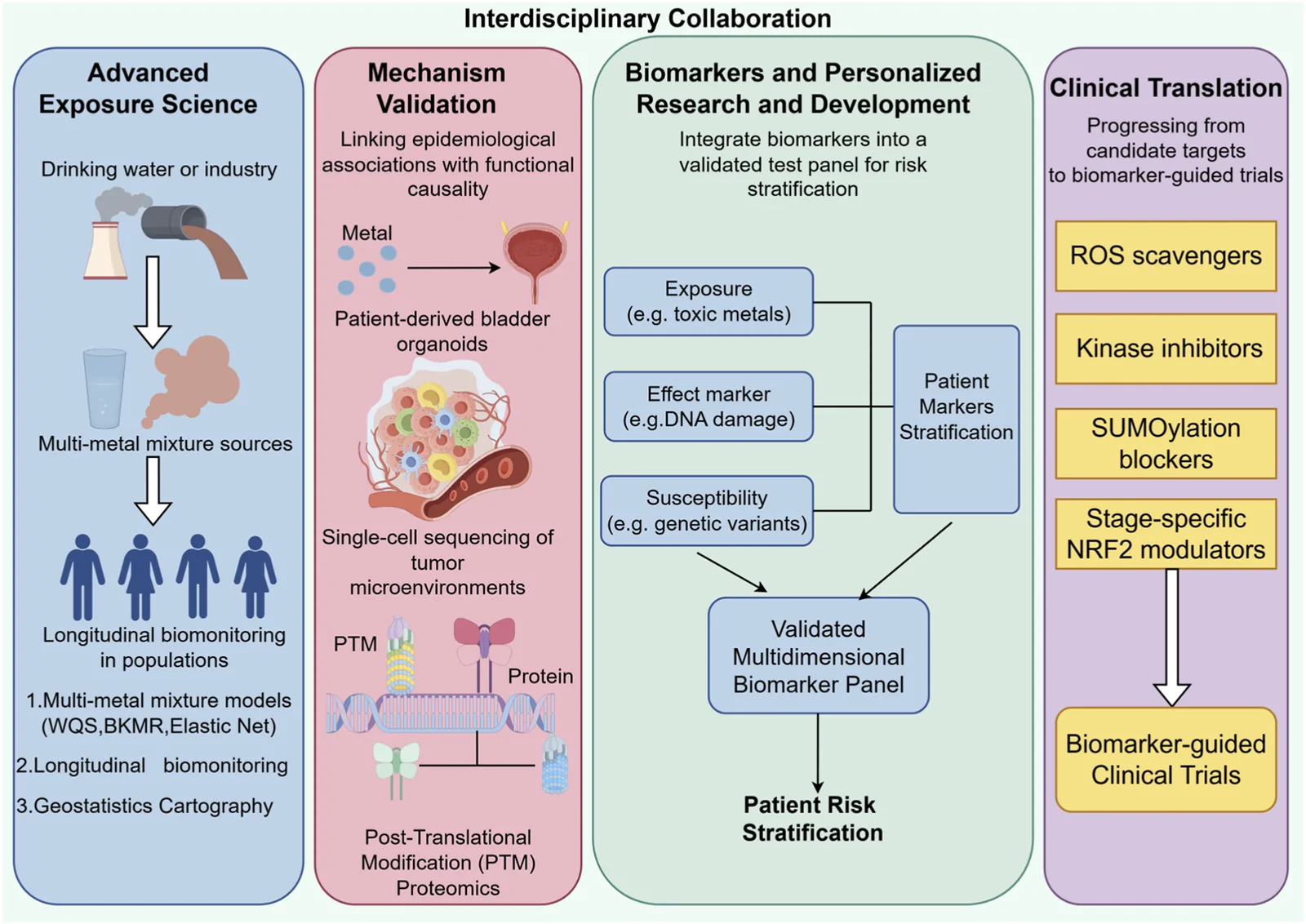
Proposed interdisciplinary roadmap for future research on toxic metal-associated bladder cancer. This schematic illustrates four interconnected research priorities (Zhang Y. et al., 2023): Advanced Exposure Science—moving from single-metal to multi-metal mixture assessment using WQS, BKMR, elastic net, and longitudinal biomonitoring (Valencia Nieto et al., 2026). Mechanistic Validation—bridging epidemiological associations to functional causality via patient-derived organoids, single-cell sequencing, and PTM proteomics (Jahrreiss et al., 2020). Biomarker Development—establishing a validated multidimensional panel integrating exposure, effect, and susceptibility markers for risk stratification (Parida and Patel, 2023). Clinical Translation—progressing from candidate targets through advanced delivery systems to biomarker-guided clinical trials. Interdisciplinary collaboration among environmental health sciences, analytical toxicology, molecular epidemiology, and urological oncology serves as the foundation, ultimately culminating in precision prevention and personalized intervention for metal-exposed populations.

### 5.1 Multi-metal Co-exposure: from single-effect to mixed-model assessment

Existing research has overwhelmingly focused on the biological effects of individual metals. Yet real-world scenarios invariably involve simultaneous exposure to complex metal mixtures. The combined effects, whether synergistic, antagonistic, or additive, remain severely understudied (El-Agrody et al., 2024). Observational studies have detected elevated concentrations of multiple toxic metals in bladder cancer tissues and have correlated them with oxidative stress markers and signaling aberrations (El-Agrody et al., 2024). However, these data do not clarify whether metals interact or how they jointly influence carcinogenic pathways. Furthermore, proteomic evidence suggests that different metals induce distinct yet overlapping protein expression profiles. This implies that simple additive models may be inadequate (Chen et al., 2020). This knowledge gap may make current risk assessments based on single-metal paradigms misleading in real-world exposure contexts.

Addressing this knowledge gap will require a concerted effort to establish more physiologically relevant mixed-metal exposure models that better recapitulate environmental and occupational scenarios. To address the challenge of metal mixtures, advanced statistical approaches that can handle high-dimensional correlated exposures and non-linear dose-response relationships are needed. In this regard, future studies must integrate advanced statistical frameworks such as Bayesian Kernel Machine Regression (BKMR)—a nonparametric method that flexibly models complex exposure-response relationships and interactions without assuming a predefined functional form—or Weighted Quantile Sum (WQS) regression—a dimensionality reduction approach that constructs a weighted index to estimate the overall mixture effect and identify major contributors—to parse the synergistic or antagonistic interactions among metal mixtures in bladder carcinogenesis. Complementing these epidemiological approaches, systematic toxicological studies employing factorial or high-throughput screening designs in bladder epithelial models could help delineate the molecular interaction networks triggered by metal mixtures. However, careful interpretation is warranted given the persistent gap between experimental conditions and real-world exposure scenarios. Integrating computational toxicology with empirical exposure data will be essential for unraveling the complex effects of combined exposures, yet achieving this integration will require sustained investment in both biomonitoring infrastructure and interdisciplinary collaboration.

### 5.2 Mechanistic validation: bridging epidemiological associations and functional causality

Epidemiological studies have consistently linked elevated metal levels to bladder cancer risk and oxidative stress markers. Mechanistic studies have identified specific signaling cascades (Panaiyadiyan et al., 2023; Yi et al., 2025). However, a profound disconnect persists between population-level correlational data and functional causality. Most mechanistic evidence derives from *in vitro* cell models or animal experiments. These often use metal doses, exposure durations, and cell types that deviate significantly from human exposure conditions (Panaiyadiyan et al., 2023). Conversely, population-based biomonitoring studies lack the biospecimens or molecular pathology data needed to validate whether pathways identified *in vitro* are operative in exposed patients. This translational gap severely limits the credibility of proposed mechanistic models and their utility for biomarker development.

To bridge this gap, future research could benefit from embedding mechanistic validation within prospective cohort designs that integrate comprehensive environmental monitoring, longitudinal biospecimen banking, and multi-omics profiling. Functional validation using patient-derived organoids or primary urothelial cells offers a promising approach to test whether metal-associated molecular aberrations observed in population samples are causally linked to malignant phenotypes; nevertheless, organoid models remain technically demanding, costly, and may not fully capture the complexity of *in vivo* tumor microenvironments. Emerging technologies—particularly single-cell sequencing and post-translational modification (PTM) proteomics—enable the analysis of oxidative stress-driven signaling dynamics at unprecedented resolution, yet their application to large-scale population studies faces challenges related to throughput, cost, and data integration. While these integrative approaches hold considerable promise for transforming correlational findings into mechanistically validated knowledge, their successful implementation will require careful experimental design, rigorous quality control, and sustained interdisciplinary collaboration.

### 5.3 Clinical translation: from candidate targets to biomarker-guided precision intervention

Significant progress has been made in identifying intervention targets, including ROS scavengers, epigenetic modulators, SUMOylation inhibitors, and NRF2 modulators. However, translating these candidate strategies into clinical applications remains in their very early stages and faces multiple intertwined practical obstacles. The stage-dependent duality of NRF2 presents a therapeutically challenging paradox: its activation is cytoprotective in early carcinogenesis but pro-tumorigenic in advanced disease (Xu et al., 2017). This temporal reversal of NRF2 function, observed primarily in experimental models, highlights a potential challenge for therapeutic targeting: indiscriminate activation or inhibition of NRF2 could produce opposing effects depending on disease stage and patient population. However, this remains a conceptual concern that requires rigorous preclinical validation and, ultimately, carefully designed clinical trials with appropriate patient stratification.

Naturally derived antioxidant candidate compounds—including lycopene, phytosterols, and Excavatolide C—demonstrate good activity *in vitro*. However, their bioavailability and delivery efficiency to target tissues are far from ideal *in vivo*. Insufficient therapeutic concentrations following systemic administration severely limit their antitumor efficacy (Yang et al., 2022; Kim et al., 2019; Wang et al., 2022). The current severe lack of rigorously clinically validated predictive biomarkers in this field is even more concerning. This makes it impossible to stratify patients or design clinical trials that enroll a targeted population to demonstrate the precise benefits of interventions. It also means the evidence base for most population-level health intervention strategies—such as lifestyle modifications and smoking cessation counseling—still relies primarily on indirect observations rather than randomized controlled trials targeting populations exposed to metals (Kozak, 2025).

Given these interlinked translational bottlenecks, future research will need to advance along multiple interdependent pathways. A priority for the field is developing a biomarker panel that integrates metal exposure metrics, oxidative damage products, and susceptibility polymorphisms. It must be rigorously validated in large-scale prospective cohorts. However, achieving this goal will require overcoming substantial hurdles, including standardizing analytical methods across laboratories, establishing reproducible cutoffs, and demonstrating clinical utility in diverse populations. The predictive performance of such a multi-biomarker panel would need to be benchmarked against established risk factors and shown to add meaningful clinical value before adoption in practice.

Regarding NRF2-targeted strategies, the stage-dependent duality of this pathway suggests that future interventions should move beyond simplistic activation-or-inhibition approaches toward a more nuanced, stage-adapted paradigm. NRF2 activators might have a role in high-risk populations with confirmed chronic metal exposure, while NRF2 inhibitors could be considered for advanced tumors with NRF2 hyperactivation. However, clinical translation of either approach will depend critically on developing and validating reliable companion diagnostics to identify patients most likely to benefit or be harmed by these interventions. Similarly, advanced delivery systems such as liposomes or polymer nanoparticles could address the poor pharmacokinetics of natural antioxidants. Still, these technologies remain in the early stages of development, and their bladder-specific targeting, long-term safety, and cost-effectiveness have yet to be demonstrated. Rigorously designed randomized controlled trials in metal-exposed populations will ultimately be needed to evaluate the benefits of dietary antioxidant supplementation, chelation therapy, and lifestyle interventions. Yet, such trials face considerable logistical, ethical, and financial challenges that should not be underestimated.

Translating mechanistic knowledge into tangible public health gains will depend on sustained collaboration across disciplines—from environmental health science and analytical toxicology to molecular epidemiology and urological oncology. By systematically addressing key bottlenecks—from biomarker validation and targeted delivery to clinical trial design and implementation-the field may gradually achieve the long-term goal of precision prevention and personalized intervention for toxic metal-related bladder cancer. However, this translation will likely require years of concerted effort and will proceed incrementally rather than through isolated breakthroughs.

### 5.4 Limitations of the search strategy and potential selection bias

While we designed a systematic and reproducible search strategy to capture the breadth of evidence on metal-associated bladder carcinogenesis, we acknowledge a potential bias toward mechanisms most extensively studied in the literature, particularly ROS-driven oxidative stress and classical oncogenic signaling pathways. This bias reflects the field’s current state rather than an intentional exclusion of alternative mechanistic frameworks. Indeed, most published experimental and epidemiological studies on heavy metal carcinogenesis have focused on oxidative stress as the primary initiating event, with relatively few investigations into mechanisms such as epigenetic reprogramming, metabolic rewiring, or tumor-stroma interactions, although emerging evidence in these areas is rapidly accumulating.

To mitigate this bias, we used a hybrid search strategy that combined structured database queries with iterative manual screening of reference lists, allowing us to include studies on less frequently explored mechanisms that the primary keyword search may not have captured. Nevertheless, the extent to which our review comprehensively represents all relevant mechanisms remains inherently limited by the publication landscape: the weight of evidence naturally skews toward well-established pathways. Future systematic reviews may benefit from bibliometric network analyses and machine learning-assisted literature mining to identify emerging or underrepresented mechanistic themes in metal carcinogenesis. Additionally, although our primary keyword search did not explicitly include terms such as “smokers,” the iterative manual screening of reference lists from epidemiological studies enabled us to capture the extensive literature on smoking as a confounder and effect modifier in metal-associated bladder cancer, as discussed in Section 2.2.

## 6 Conclusion

In conclusion, applying the Bradford Hill criteria to the synthesized epidemiological and mechanistic evidence yields a clear hierarchy of causal inference: arsenic is established as a causal risk factor for bladder cancer, with consistent associations, robust dose-response relationships, temporal precedence, and strong biological plausibility. Cadmium is likely a causal risk factor, though its independence from smoking requires further clarification through mediation analyses. Chromium is a probable risk factor, with tissue-based evidence supporting causality but serum-based prospective findings introducing uncertainty. Lead is a possible risk factor, with the weakest and most conditional evidence. This graded causal framework provides a foundation for prioritizing prevention efforts—targeting arsenic and cadmium as the most actionable environmental risks—while guiding future research to resolve uncertainties surrounding chromium and lead. Translating this framework into clinical practice will require overcoming critical bottlenecks: validating experimentally derived pathways in exposed populations, developing reliable multi-biomarker panels, and conducting rigorously designed trials of targeted interventions. Achieving this will demand sustained interdisciplinary collaboration to bridge environmental exposure science and precision oncology.
